# Investigation of the Contact Interface between Natural Fibre Metal Laminates under Tension Using Finite Element Analysis (FEA)

**DOI:** 10.3390/polym14214650

**Published:** 2022-11-01

**Authors:** Chun Han Song, Khaled Giasin, Abu Saifullah, Antigoni Barouni

**Affiliations:** Advanced Polymers and Composites (APC) Research Group, School of Mechanical and Design Engineering, University of Portsmouth, Portsmouth PO1 3DJ, UK

**Keywords:** fibre metal laminates, natural fibre composites, progressive damage analysis, failure mechanism, finite element analysis, numerical analysis, tension, delamination

## Abstract

Fibre Metal Laminates (FMLs) consist of layers of metals combined with layers of fibre-reinforced composites bonded together to create a laminate. The behaviour of a Fibre Metal Laminate (FML) with natural fibre composites has been investigated in this study with a specific focus on the performance of the laminate under uniaxial tension. The integration of aluminium layers with natural fibre flax/pp layers at different fibre orientations has been numerically modelled and analysed, by investigating the contact interface between natural fibre metal laminates (NFML) using finite elements (FE) implemented in ABAQUS/Explicit. The finite element model was developed by the isotropic-hardening behaviour of metal layers, the built-in Hashin damage model and cohesive surface-based behaviour for the interface. The results of the simulation included stress–strain response, failure sequences, delamination effect and ultimate tensile strength. It was found that those results are significantly affected by the layup sequence, giving a significant advantage to the unidirectional laminate, when the uniaxial loading is taken into consideration. This advantage is measured as a 41.9% reduction of the ultimate tensile strength when the flax fibres are oriented at [0/90] configuration between the aluminium layers and a 30% reduction when the fibres are oriented at [±45] angles.

## 1. Introduction

FMLs have become very common in the aerospace industry during the past decades. It is a hybrid material that is built up from metal layers and fibre-reinforced composite layers, taking advantage of the benefits of the metallic layers, such as their high bearing strength and impact resistance, as well as the good fatigue resistance and high stiffness-to-weight ratio of the composites [1,2]. The main benefit of the FMLs is sourced from the bridging effect between the composite layer and metal layer, which can significantly slow down the fatigue crack growth rate. The bridging effect is taking place between the composite layer and the delamination plane. When the metal layer cracks, the composite layer will continue to hold and reduce the stress intensity [3,4,5,6]. Hence, the stress concentration will reduce, which can lead to a slow crack growth rate. This improved fatigue behaviour of FMLs makes them ideal for aircraft structures. Asundi et al. [7] found that using glass fibre-reinforced aluminium laminates as a material of the fuselage skin can reduce the weight by 15–25% without compromising the performance. The most successful application is used as the upper fuselage skin material of the Airbus A380. FMLs in aerospace applications might experience constant tensile and compressive loading [8], making the mechanical and failure behaviour of FMLs under tension very critical, which can include matrix cracking, fibre breakage and debonding of layers [9].

Over the years, the fibre-reinforced composite materials have been used in a variety of engineering applications and domains, where the tensile fatigue behaviour is investigated for glass fibre laminates [10], and the compressive strength is improved on glass fibre composites by winding additional layers around the longitudinal glass fibres [11], and the development of data-driven models and neural network models has been attempted for the prediction of the flexural performance of fibre-reinforced concrete columns [12,13]. Various attempts have taken place in order to investigate the performance of fibre metal laminates under different loading scenarios such as bending, tensile, compression, impact and shear [14]. Khalili et al. [15] investigated the mechanical properties of fibre metal laminates with different lay-ups of laminates and metal layers by three different testing scenarios: bending, impact and tensile. The results indicated that the damage tolerance limit and energy absorption of FMLs are superior to fibre-reinforced composites. Detailed work has been done by Sharma et al. [16] on quasi-static testing of titanium-based glass fibre-reinforced laminates. The tensile behaviour of four different layups was evaluated. The overall thickness of fibre metal laminates was fixed while changing the position and thickness of the metal layers in the stack. From the results, the layup sequence rarely affected the initial modulus of the fibre metal laminates. Moussavi et al. [17] combined classical laminate theory with the elastic-plastic behaviour of the aluminium layer to predict the stress–strain behaviour of fibre metal laminates. The fibre metal laminates model was loaded by uniform tensile force along the fibre direction while no cohesive behaviour was considered between any two layers. They concluded that FMLs with zero orientation fibre layer show improvement in maximum tensile strength. In another experiment, Hashemi et al. [18] investigated the performance of 3D glass fibre-based fibre metal laminates under tensile loading, when the laminates were manufactured under a different set of parameters (i.e., temperature, pressure and time), with the pressure contributing more to the mechanical properties of the laminate.

Numerical simulations are increasingly being used to study the performance of fibre metal laminates. Soltani et al. [19] simulated the tensile behaviour of FMLs under in-plane loading using Finite Element (FE) modelling, proposing a model able to analyse GLARE laminates in structural applications. Sharma et al. [20] investigated the tensile response of FMLs using three different specimens with constant volume but different configurations of metal and composite layers. Stress–strain curves of the FMLs were presented and indicated the failure stage of each specimen. It was stated that the configuration of metal and laminate layers would affect the tensile behaviour of the FML, where the existence of a metal layer between the composite layers demonstrates weaker performance than without a metal layer between the composite layers. A work that discusses the progressive damage and failure mechanisms of open-hole fibre metal laminates was done by Du et al. [21]. The performance of three different carbon fibre-reinforced PEEK prepregs layups, namely unidirectional, cross-ply and quasi-isotropic layups, was investigated. It was found that the failure of the FMLs began from fibre breakage. After the laminates met the yield point, the fibres would break first, followed by the failure of the matrix. The facture of metal layers took place once the delamination entirely occurred between the metal and laminate layer. Furthermore, higher-order shear deformation theory has been implemented to predict the shear strains through the thickness of composite laminated plates, with the use of FE modelling [22] as well as 3D deformations and progressive failures have been predicted for conventional fibre metal laminates using a user subroutine [23].

In the last few years, the production of sustainable products has gained more attention from manufacturers and researchers, leading them to integrate natural fibre composites into different applications [24,25]. Natural fibre composites are lightweight, easier to produce from natural resources and exhibit reduced environmental impact compared to synthetic fibres, making them a very promising replacement to synthetic fibres in demanding applications, such as automotive [26]. Fidelis et al. [27] investigated the tensile strength of several natural fibre composites, including jute, sisal, curaua, coir and piassava, concluding that the curaua fibres have the highest young modulus and tensile strength among other natural fibres. The use of natural fibre-reinforced composites has been increasingly used in semi-structural applications, where impact toughness is important [28], as well as in more demanding applications with the cycle loading performance of the material is critical [29]. However, the use of natural fibres is still limited to applications where the demand for structural integrity is high and critical, such as aerospace. The combination of natural fibre composites with metallic layers, to provide a sustainable alternative to FMLs, could replace the conventional FMLs in aerospace and improve the carbon footprint of the structures significantly, bringing a novelty to aerospace materials. Several implications can appear during this attempt, with the highest would be the compatibility of the two materials, with a greater focus on the delamination between the metallic and composite layers. Therefore, a preliminary numerical study is essential to understand the behaviour of these novel materials.

This paper aims to perform a finite element analysis for simulating a natural fibre metal laminate (NFML) composite structure and its failure behaviour under tension. Hence, the main focus is to study the damage initiation and damage evolution of the composite structure under tensile loading. In the simulation, cohesive-surface-based behaviour was applied to observe the delamination and the Hashin 2D criterion was involved to capture the damage initiation and evolution in the composite. Three types of layup configurations were considered, such as unidirectional, cross-ply and quasi-isotropic, to study the effect of fibre orientation on the failure modes, which is an important factor affecting the strength of the composite. The results obtained from the simulation such as the stress distribution and damage evolution of each layer will be discussed in the results section. 

## 2. Materials and Methods

### 2.1. Metallic Layers

The metallic layer used in this study is Aluminium 2024-T3. The elastic and plastic behaviour of the metallic layer was modelled by using isotropic hardening. The isotropic hardening data for Al 2024-T3 are listed in Table 1 [20].

### 2.2. Composite Layers

Two different composite materials were involved in this study; E-glass/Epoxy composite laminates and flax/polypropylene composite. E-glass/Epoxy composite laminates were modelled in the first part of the simulation to validate the developed FE model using the work of Sharma [20] and the properties are shown in Table 2. The second part of this study investigated the performance of natural fibre flax/polypropylene composite. The material properties used for natural fibre flax/polypropylene composite are listed in Table 3 [30].

For the prediction of the damage initiation and evolution, a cohesive-surface-based behaviour method was implemented, using the material properties summarised in Table 4 [30].

### 2.3. Configuration of NFMLs

To validate the developed FE model in this work, a validation study was initially conducted with already existing literature. The fibre metal laminate with glass fibre composite layers, named GLARE 3/2-0.4, was used for the initial validation study [19].

#### 2.3.1. Glare 3/2-0.4 

**Figure 1:**
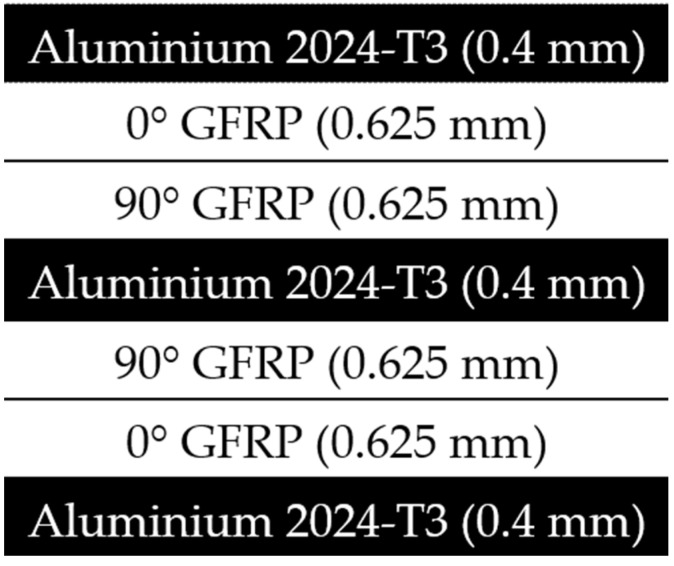
Schematic diagram of GLARE 3/2-0.4.

The Glare3/2-0.4 laminate was made of three sheets of aluminium 2024-T3 alloy alternating with two layers of E-glass/Epoxy composite laminates. The schematic diagram of the specimen is shown in Figure 1. Due to the symmetry of the geometry and loading of the specimen, only a quarter of the model was simulated. The total thickness of the specimen is 3.7 mm, while the aluminium thickness is 0.4 mm each and the composite layer is 0.625 mm each.

#### 2.3.2. Aluminium/Flax Fibre Metal Laminates 

**Figure 2:**
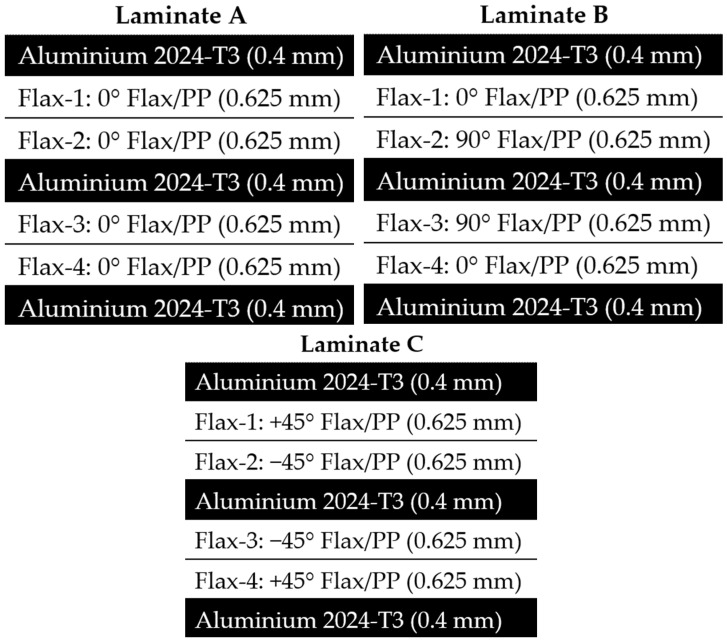
The various layup of Aluminium/Flax fibre metal laminates.

The main work for this study was conducted on a natural fibre metal laminate (NFML), where flax-fibre-reinforced laminates were used combined with Aluminium layers. The material is subjected to tensile loadings at one end of the specimen. The effect of lay-up configuration on fibre metal laminates with flax fibre reinforcement is subsequently investigated in this study. Three different laminate configurations were used for this investigation, where three aluminium layers and two embedded flax-fibre/polypropylene composite layers were used for each configuration. The laminate configurations used include unidirectional, cross-ply and biaxial configurations. The thickness of the aluminium and flax/pp layers is listed in Table 5. The schematics of the fibre metal laminates with different stacking sequences are presented in Figure 2. The size and geometry of the model remain the same as described in ASTM D3039.

## 3. FE Modelling Approach

**Figure 3:**
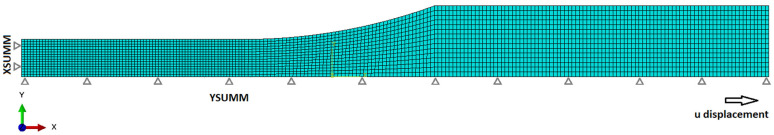
The FE model of the quarter dog-bone specimen.

For the investigation of the tensile behaviour of NFMLs, an FE model was developed using ABAQUS/Explicit, using the geometry as described in the experimental testing standard ASTM D3039. The dog-bone-shaped geometry was used where one-quarter of the geometry was being modelled, due to symmetry reasons, as shown in Figure 3.

FMLs will undergo different types of failure modes during the deformation process, such as delamination between two layers, fibre breakage, fracture of the metal layer, and the debonding process in the laminates layer and matrix cracking [31]. The cohesive surface-based behaviour was used for this numerical modelling to define the interaction between two surfaces. The cohesive-surface-based behaviour method uses traction-separation theory to define cohesive interaction between two surfaces without having cohesive elements in the geometry.

Traction-separation behaviour involves three stages, namely, the linear elastic behaviour, the damage initiation and the damage evolution. Linear elastic behaviour with uncoupled traction separation was used, meaning shear direction cohesive forces are not affected by pure normal separation and normal direction cohesive forces are not affected by pure shear slip. Hence, the uncoupled behaviour relation is written as follows.

(1) $$t=\left\{\begin{array}{c} t_{n}\\ t_{s}\\ t_{t} \end{array}\right\}=\left[\begin{array}{ccc} K_{nn} & 0 & 0\\ 0 & K_{ss} & 0\\ 0 & 0 & K_{tt} \end{array}\right]\left\{\begin{array}{c} \delta_{n}\\ \delta_{s}\\ \delta_{t} \end{array}\right\}$$

where $t_{n}$, $t_{s}$, $t_{t}$ indicate the nominal traction vectors in the orthogonal direction. The value of penalty stiffness for the traction separation behaviour is denoted as *K*, and relative displacement is denoted as δ. Both are involved in the orthogonal direction.

Damage initiation represents the beginning of the degradation of the contact surface under loading conditions. The degradation of the material starts when the contact stresses meet the damage initiation criterion defined by the user. A value of 1 in the damage initiation criterion represents the failure of the contact surface. The quadratic stress criterion was chosen for this simulation. It involves a peak value of the contact stress to determine the degradation.

(2) $${\left(\frac{t_{n}}{t_{n}^{0}}\right)}^{2}+{\left(\frac{t_{s}}{t_{s}^{0}}\right)}^{2}+{\left(\;\frac{t_{t}}{t_{t}^{0}}\right)}^{2}=1$$

where $t_{n}^{0}$, $t_{s}^{0}$ and $t_{t}^{0}$ represent the interface strength parameter in the orthogonal direction.

Damage evolution indicates the degradation of the material, which is cohesive stiffness after fulfilling the initiation criteria. The damage evolution is according to softening laws. Either displacement or fracture energy can determine the softening laws for simulating the delamination. The Benzeggagh–Kenane fracture criterion is applied to the simulation by assuming the same value of fracture energy in the first and second shear direction ($G_{s}^{C}=G_{t}^{C})$. All parameters of the cohesive-surface-based behaviour method used in this study were summarized in Table 2.

To predict the behaviour of composite layers, a constitutive model is used to describe the response of linear elastic behaviour of fibre-reinforced material. The relationship between stress and strain before damage initiates is defined as $\sigma =C_{d}\varepsilon$. The damaged elasticity matrix replaces the elasticity matrix once damage initiation happens. The damaged elasticity matrix $C_{d}$ is given below.

(3) $$\left[C_{d}\right]=\left[\begin{array}{ccc} \left(1-d_{f}\right)E_{1} & \left(1-d_{f}\right)\left(1-d_{m}\right)v_{21}E_{1} & 0\\ \left(1-d_{f}\right)\left(1-d_{m}\right)v_{12}E_{2} & \left(1-d_{m}\right)E_{2} & 0\\ 0 & 0 & \left(1-d_{s}\right)G_{12}D \end{array}\right]$$

(4) $$D=1-\left(1-d_{f}\right)\left(1-d_{m}\right)v_{12}v_{21}$$

(5) $$d_{f}=d_{f}^{t}\;\;if\;\sigma_{11}\geq 0,\;or\;d_{f}^{c}\;\;if\;\sigma_{11}<0$$

(6) $$d_{m}=d_{m}^{t}\;\;if\;\sigma_{22}\geq 0,\;or\;d_{m}^{c}\;\;if\;\sigma_{22}<0$$

(7) $$d_{s}=1-\left(1-d_{f}^{t}\right)\left(1-d_{f}^{c}\right)\left(1-d_{m}^{t}\right)\left(1-d_{m}^{c}\right)$$

where the subscripts f, m, s represent fibre, matrix and shear, respectively, and the subscript d is damage. $E_{1}$ and $E_{2}$ are Young’s modulus in the fibre and matrix direction, respectively, $G_{12}$ and $v_{12}$ are the shear modulus and Poisson’s ratio in the X-Y direction.

Equation (3) above defines how the damage of fibre and matrix changes the material’s stiffness according to four different failure modes, as described in the 2D Hashin failure criterion. ABAQUS offers the Hashin 2D criterion to predict the four different failure modes of the shell element. Failure of the composite is defined as the element reaching the yield stress and leading to damage occurrences. Maximum stress or strain theory will be needed to determine the initial damage to the matrix or fibre [32]. The purpose of using the Hashin-based damage criterion is to evaluate different failure modes in a different direction [33]. The general form of four different damage initiation modes is defined below.

$$\mathrm{Fibre}\;\mathrm{tension}\;\sigma_{11}\geq 0$$

(8) $$F_{f}^{t}={\left(\frac{\sigma_{11}}{X^{t}}\right)}^{2}+\alpha {\left(\frac{\sigma_{12}}{S^{L}}\right)}^{2}$$

$$\mathrm{Fibre}\;\mathrm{compression}\;\sigma_{11}<0$$

(9) $$F_{f}^{C}={\left(\frac{\sigma_{11}}{X^{C}}\right)}^{2}$$

$$\mathrm{Matrix}\;\mathrm{tension}\;\sigma_{22}\geq 0$$

(10) $$F_{m}^{t}={\left(\frac{\sigma_{22}}{Y^{t}}\right)}^{2}+{\left(\frac{\sigma_{12}}{S^{L}}\right)}^{2}$$

$$\mathrm{Matrix}\;\mathrm{compression}\;\sigma_{22}<0$$

(11) $$F_{m}^{C}={\left(\frac{\sigma_{22}}{2S^{t}}\right)}^{2}+\left[{\left(\frac{Y^{C}}{2S^{T}}\right)}^{2}-1\right]\left(\frac{\sigma_{22}}{Y^{C}}\right)+{\left(\frac{\sigma_{12}}{S^{L}}\right)}^{2}$$

where $F_{f}^{t}$, $F_{f}^{C}$ indicate failure mode of fibre in tension and compression; $F_{m}^{t}$, $F_{m}^{C}$ indicate the failure mode of the matrix in tension and compression. $\sigma_{11}$, $\sigma_{22}$ and $\sigma_{12}$ represent the effective stress tensor. $X^{t}$ and $X^{C}$ are the tensile strength and compressive strength of laminate in the longitudinal direction while $Y^{t}$ and $Y^{C}$ are the tensile strength and compressive strength of laminate in the transverse direction. $S^{L}$ and $S^{T}$, respectively, represent the shear strength of the laminate in the longitudinal and transverse directions.

Due to the symmetry of the geometry and loading of the specimen, only a quarter of the specimen was modelled. The FE model is subjected to tensile loadings at one end of the specimen, whereas encastre boundary conditions were applied on the other end. The built-in fibre-reinforced material damage model will be used in this simulation. A mesh convergence study was performed to determine the minimum element size. ABAQUS offers general contact in ABAQUS/Explicit solver, where each interface of two layers was defined. For the interface between metal and composite layers, the friction of 0.3 was used, while a friction value of 0.5 was used as input for the interface between the composite layers [34]. The element type used for the metal layer was eight-node solid brick elements (C3D8R) and for the composite layer was the eight-node continuum shell element (SC8R). ABAQUS explicit option was selected for this simulation as it is more suitable for highly nonlinear analysis and can reduce the required time. In the GLARE 3/2-0.4 FE model, no damage evolution was applied. However, the model for aluminium/flax fibre metal laminates does involve a ductile damage model. Once the damage initiation has been satisfied, Hillerborg’s fracture energy damage evolution criterion will be introduced. This ductile damage model includes the fracture strain and fracture energy to modify the failure of metal. 

## 4. Results and Discussion 

**Figure 4:**
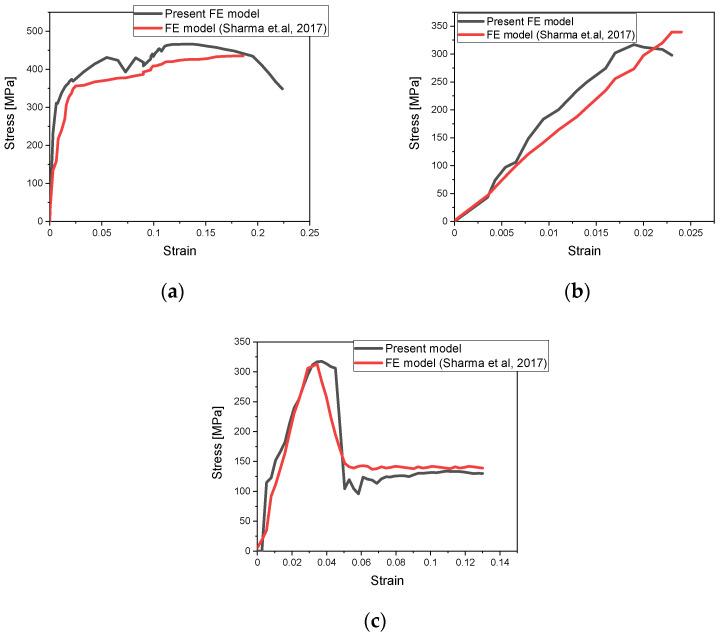
Stress–strain curves of Present FE model and Sharma et al. FE model [20] for (**a**) AL-2024 T3 alloy; (**b**) of [0°/90°/90°/0°] GFRP; (**c**) GLARE 3/2-0.4.

To initially validate the developed FE model, the strain–stress curves were compared to the results presented in [20] for three different material types: (a) a 0.4 mm thick aluminium plate, as shown in Figure 4a, (b) a cross-ply GFRP laminate, as shown in Figure 4b, (c) a GLARE 3/2-0.4 laminate, as shown in Figure 4c. All three curves exhibit very good agreement with the current FE model. Also, by comparing the ultimate tensile strength, the percentage error is 0.958%. As can be seen, all curves agree very well, especially around the initiation of delamination, a fact which enhances the use of the current FE model for further simulations. 

The stress–strain curves, failure sequences, delamination effect, ultimate tensile strength and effect of composite layup configurations will be discussed in this section.

**Figure 5:**
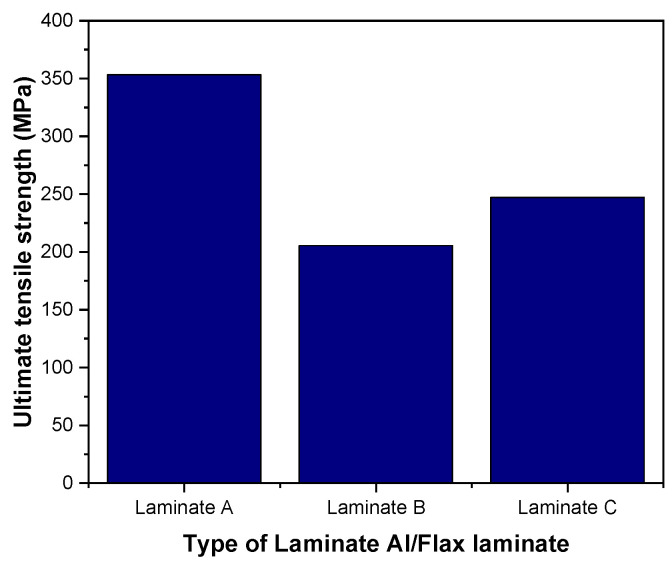
Comparison of ultimate tensile strength of the three laminate configurations.

**Figure 6:**
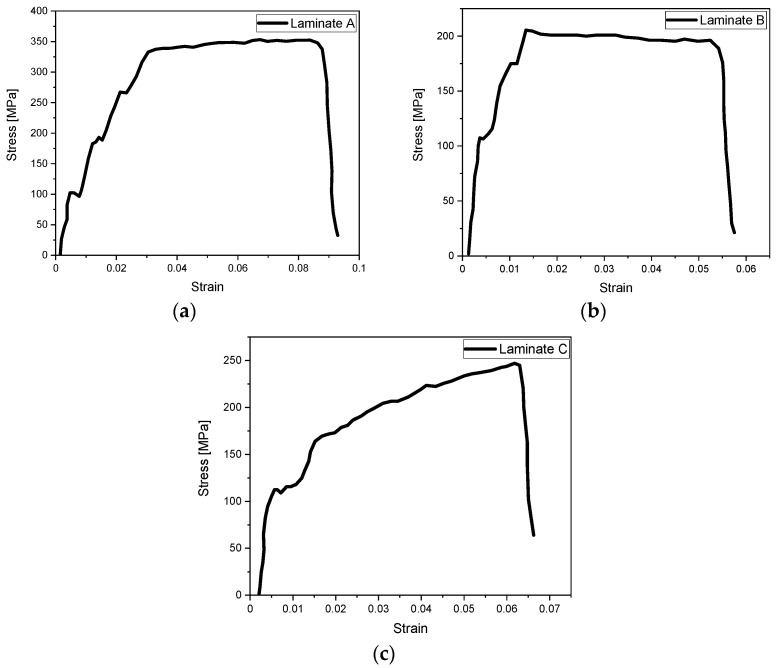
Stress–strain curves of (**a**) laminate A; (**b**) laminate B; (**c**) laminate C.

The stress–strain response for each configuration A, B and C, under tensile loading is described in Figure 5 and Figure 6. According to Figure 5, laminate A with unidirectional fibre orientation has the highest tensile strength, followed by laminate C, and laminate B exhibits the lowest UTS among the chosen laminates. Specimen A has the highest tensile strength mainly due to the fibre orientation being aligned to the tensile load in the longitudinal direction. Figure 5 shows a significant difference in ultimate tensile strength when changing the layup of the lamina. For example, laminate B with a cross-ply configuration demonstrates a reduction of 41% of ultimate tensile strength compared to laminate A. 

Due to the same metal distribution and metal volume, the curves of the three specimens in Figure 6 look similar but fail at different strain values. For laminate A, at the beginning of loading, the maximum stresses are mainly concentrated on the aluminium layers and only appear on the edges of the composite layers. The stress slowly grows into the whole structure and is distributed evenly. This is because of the characteristics of 0°-degree fibre orientation which increases the tensile loading capability of the laminate. At this point, the longitudinal stresses on the laminate will continue to rise and reach the ultimate tensile strength. Once the ultimate tensile strength is reached, cracks will appear in the metal layer and the whole laminate will fail.

**Figure 7:**
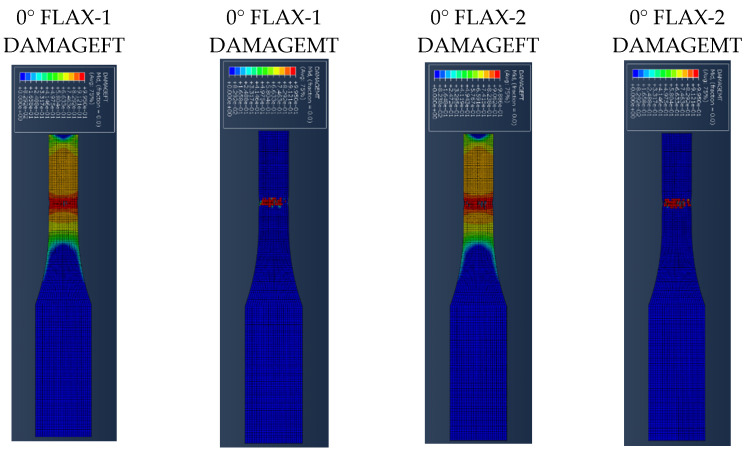
Damage evolution for fibres and matrix of top composite layers of Laminate A at 2.6 mm displacement.

**Figure 8:**
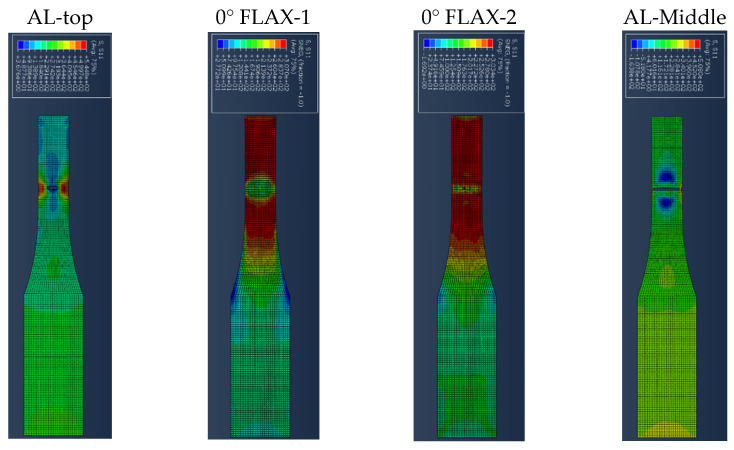
Stress distribution of the four top layers of laminate A at 2.8 mm displacement.

**Figure 9:**
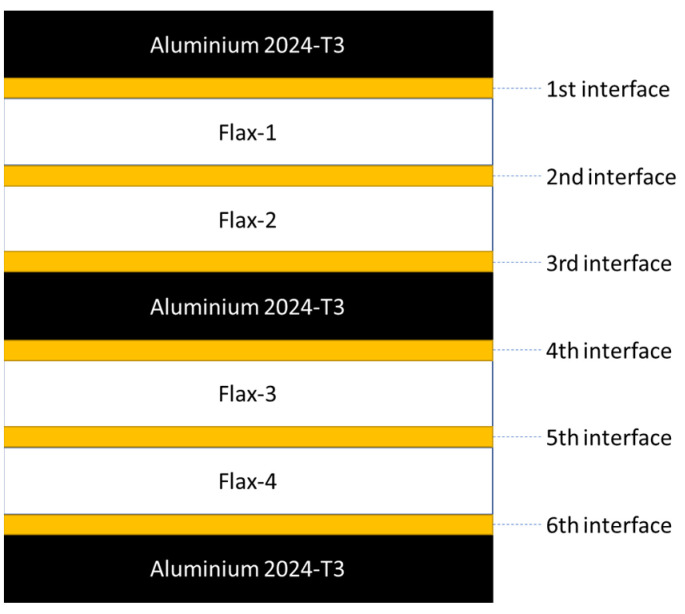
Schematic of interfaces between layers of the NFML.

**Figure 10:**
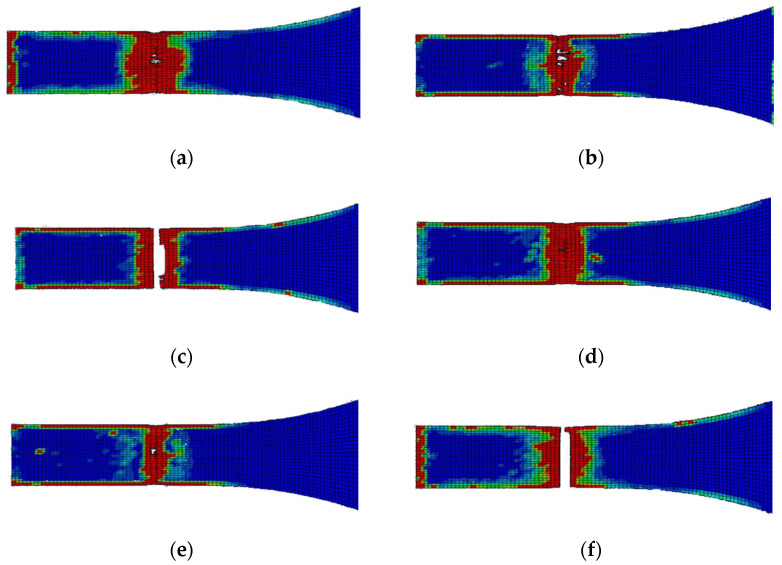
Delamination of each layer of laminate A after failure; (**a**) 1st interface; (**b**) 2nd interface; (**c**) 3rd interface; (**d**) 4th interface; (**e**) 5th interface; (**f**) 6th interface.

Figure 7 presents the results for the damage evolution in both matrix and fibres in tension for each of the composite layers of laminate A at 2.6 mm displacement, with the naming of the flax layers following the convention of Figure 2. The damage evolution started with the matrix cracking that was first initiated at both 0° flax composite layers, as shown in Figure 7. At the same time, the fibres of both 0° flax composite layers are still capable of holding stress due to their characteristics. After the matrix failure, the fibre starts to break. This led to the failure of the specimen. As shown from the stress distribution in Figure 8, at 2.8 mm displacement, the middle aluminium layer has already failed, and cracking starts to appear at the 0° Flax-2 layer too. For the interfaces between the layers, which are numbered in Figure 9, the delamination at the interface between each layer after the failure of the specimen is shown in Figure 10. The red-coloured areas in these contours represent the highly delaminated areas of each interface (with a value of 1.0 of the damage indicator) whereas the blue-coloured areas indicate no delamination (value 0.0 of the damage indicator). The edges of the layer between the top aluminium and bottom aluminium layers have less delamination than those in between the two composite layers and mid-aluminium layers. 

**Figure 11:**
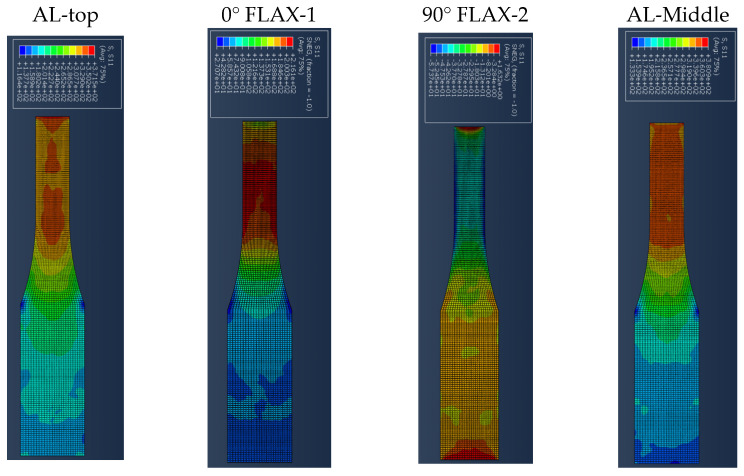
Stress distribution of each top layer of laminate B at 0.5 mm displacement and.

**Figure 12:**
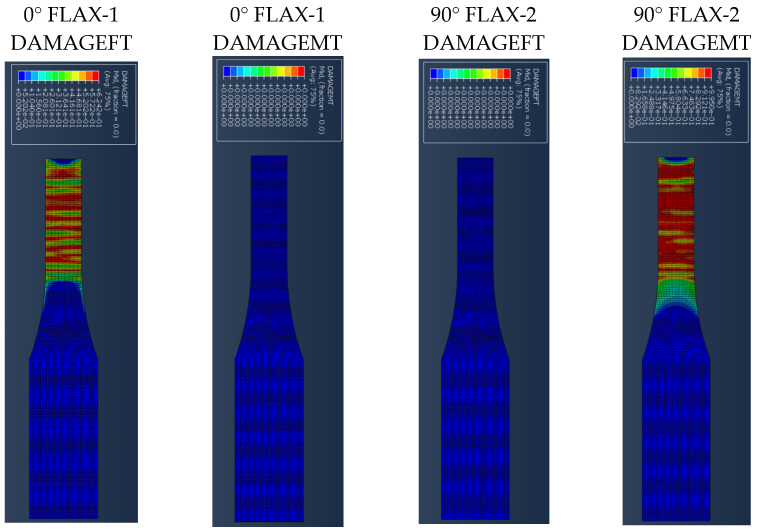
Damage evolution of each composite layer of the laminate B at 2 mm displacement.

The stress distribution of laminate B is mainly focused on the 0° ply and aluminium layers as shown in Figure 11 at a displacement of 0.5 mm. The longitudinal stress carried by the aluminium layer and 0° ply is around 371 MPa and 216 MPa, respectively, while the 90° ply only carried 1.6 MPa of stress, due to the matrix cracking occurring at the 90° ply during the early stage of tension loading. With further loading, the propagation of matrix cracking will be distributed across the 90° ply. The fibres belonging to the 0° ply will start to break, and the direction of propagation is perpendicular to the fibre direction, as seen in Figure 12.

**Figure 13:**
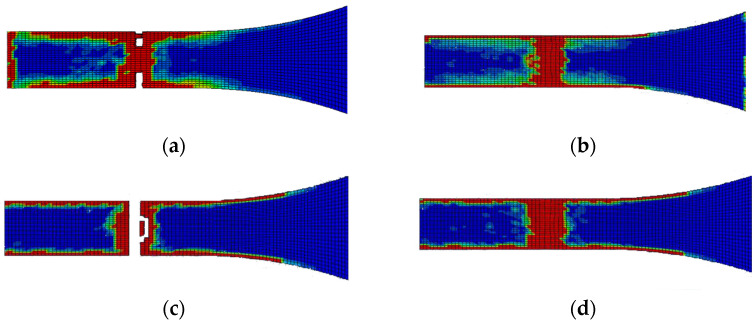

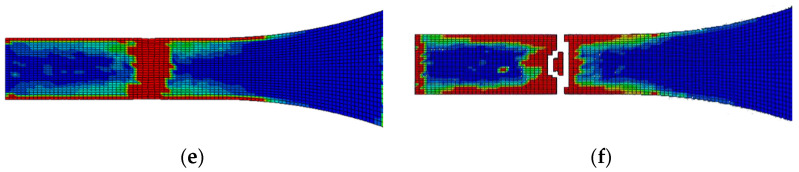
Delamination of each layer of laminate B after failure; (**a**) 1st interface; (**b**) 2nd interface; (**c**) 3rd interface; (**d**) 4th interface; (**e**) 5th interface; (**f**) 6th interface.

The edge delamination formation occurred first between the 90° flax composite layer and the mid-aluminium layer of laminate B (Figure 13c). Unlike laminate A, the delamination happens randomly of the edges during the early stage. After the delamination occurred on the edges of the 90° flax composite and mid-aluminium layers, it started to distribute into adjacent layers. The 1st and 6th interfaces have delamination on the end edge of the laminate while other interfaces do not, as shown in Figure 13. Again here, the red-coloured areas in these contours represent the highly delaminated areas of each interface (with a value of 1.0 of the damage indicator) whereas the blue-coloured areas indicate no delamination (value 0.0 of the damage indicator). The area of delamination across interfaces 1 and 6, that is, between Al and 0° composite layer layers, is larger than the delamination observed across interfaces 3 and 4, that is, between Al and 90° composite layers. It can be concluded that the primary failure starts from matrix cracking of 90° ply, and fibre tension damage in the 0° layer occurred during the early stage of loading. Once the matrix cracking of 90° ply started, the 90° ply lost its ability to withstand the stress. Then the fibre of the 0° ply and aluminium layer will be responsible for holding the stress. Once it reached the ultimate strain, the specimen failed.

**Figure 14:**
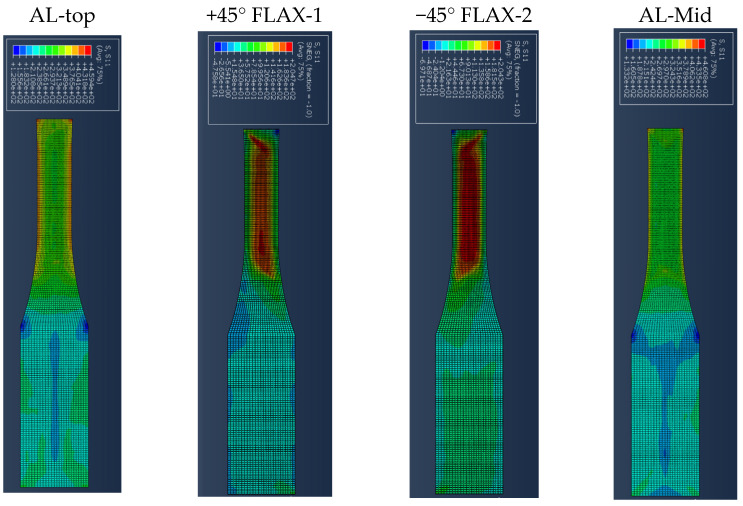
Stress distribution of each top layer of laminate C at 3 mm displacement.

**Figure 15:**
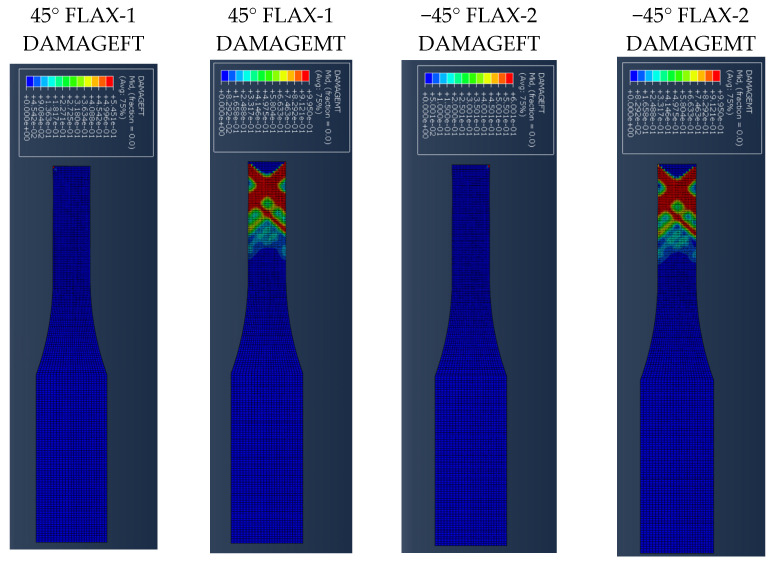
Damage evolution of each top composite layer of specimen C at 1 mm displacement.

The stress distribution of laminate C follows the fibre orientation of the composite layer. Figure 14 shows the displacement at 3 mm, where the stress concentration of the 45° flax layer and the −45° flax layer is aligned to the fibre direction. Figure 14 shows that the stresses at the aluminium layers are concentrated around the edges. It is worth observing that, when the angle of the principal loading direction is more than 45 degrees compared to the fibre orientation, the metal layers are dominating the load carrying for the whole FML, which can be also verified from Figure 14, with the stresses developed on the Al layers being around 460 MPa, as opposed to circa 205 MPa for the composite layers. The results presented here show that the aluminium layers carry almost double the magnitude of stress under tensile loading compared with the composite layers. Figure 15 shows the different damage modes developed on the flax layers of the NFML. It is evident that the matrix cracking is the first model to be observed and is extended along the ±45° direction, as expected.

**Figure 16:**
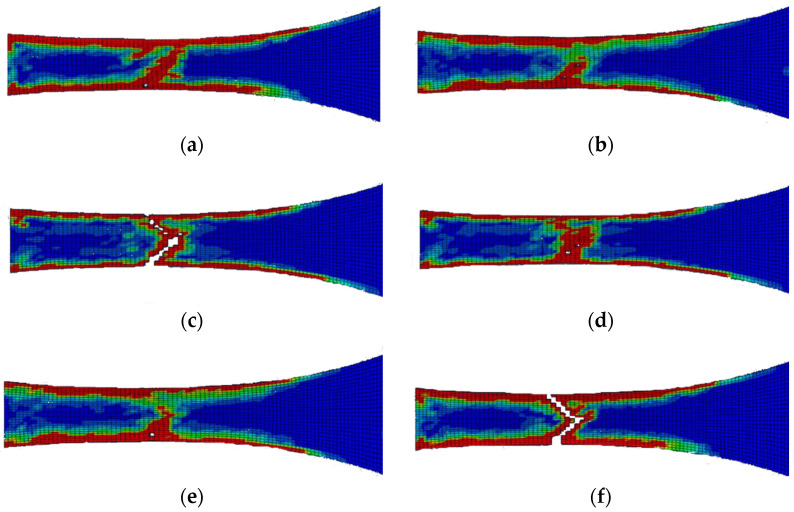
Delamination of each layer of laminate C after failure; (**a**) 1st interface; (**b**) 2nd interface; (**c**) 3rd interface; (**d**) 4th interface; (**e**) 5th interface; (**f**) 6th interface.

Unlike laminate B, the delamination of laminate C does not have sequences. It rapidly grows on the edge of the specimen. Figure 16 shows that the delamination occurs at the failure area and follows the 45° fibre orientation pattern. The red-coloured areas in these contours represent the highly delaminated areas of each interface (with a value of 1.0 of the damage indicator) whereas the blue-coloured areas indicate no delamination (value 0.0 of the damage indicator). The damage evolution of laminate C, starting with matrix damage, occurred at both 45° and −45° flax layers. Fibre breakages happened when the specimen failed. The failure of the specimen also follows the fibre orientation.

**Figure 17:**
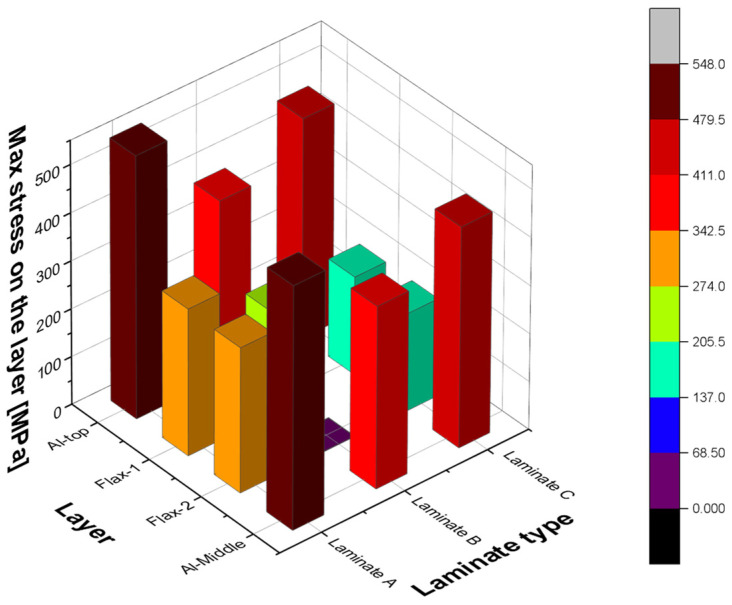
Maximum stress comparison between all three laminate types for the first four layers of each laminate.

In summary, the three laminate configurations with different layup orientations are being investigated in this section when loaded under tension, where it was found that the stress distributions of each laminate are greatly affected by fibre orientation. For example, all layers of laminate A are capable of carrying load due to the longitudinal direction, while the 90° flax layer of laminate B does not contribute to the longitudinal loading. In addition, the stress distribution of laminate C is along either 45° or −45° fibre orientation. The maximum stresses developed in each laminate are shown in Figure 17, where the first 4 layers are presented, that is, the top and middle aluminium layers as well as the flax layers in between (Figure 9), with different orientations for each laminate. It is evident from this bar chart that both the aluminium layers are carrying most of the load in all three laminates, with the 0° flax layers being mostly loaded, as compared to the other orientations. It is also interesting to observe that the 90° fibre orientation carries the lowest load, due to being perpendicular to the loading direction. Delamination often occurs on the edges of the structure and the breakage area but only the interface between the 0° fibre and aluminium layers shows delamination on the fixed end side.

## 5. Conclusions

In this paper, the performance of fibre metal laminates reinforced with natural fibre composite layers (NFMLs) under tension is investigated and studied under different laminate configurations using an FE model developed in Abaqus. The importance of understanding fully the behaviour of these novel materials is essential, leading to their wider adoption into secondary and primary engineering applications. The combined benefit of the proposed NFMLs in terms of their lower carbon footprint and their overall reduced CO_2_ emissions has motivated this study to provide further insight into their performance. The approach used in this paper comprises a selected set of laminate configurations combining Aluminium layers with flax fibre-reinforced composite layers at different fibre orientations, in order to investigate the effect of the layup on the tensile behaviour of the material. The study is particularly focused on the contact interface between the natural fibre composite and the metal layers under tension loading. The finite element modelling approach is meso-level, which means each layer is modelled individually. The meso-level modelling approach is sufficient to analyse the behaviour of layers and the interaction between them. The developed FE model used the Hashin-2D built-in damage of composite layer and cohesive surface-based criterion in Abaqus. The damage in the interface and failure mechanisms of the fibre metal laminates was discussed in the results part of aluminium/flax fibre metal laminates. The damage to the interface, such as delamination, was also discussed and related to the fibre orientation. Three different types of layup sequences were investigated in this study and showed a different behaviour under tension loading, with the Laminate A exhibiting a higher toughness in tension, due to the fibre orientation. As expected, the fibre orientation plays a critical role in the strength and performance of the composite laminate, with the unidirectional laminate performing better under tension. However, the delamination in the interfaces of the unidirectional laminate (Figure 10) exhibits a greater extend compared to the cross-ply laminate (Figure 13). The debonding between the metallic and the composite layers is of great importance and it’s evident from the stress distribution analysis of all three laminates examined in the present study that the aluminium layers are mainly responsible for carrying a greater load compared to the composite layers.

The limitations observed in this study are related to the prediction of the full stress state of the laminate, due to the use of the 2D Hashin model. However, the results produced are of great importance, as they offer an initial understanding of the performance of the novel materials. The feasibility of this study could be further improved in a future paper, to accommodate a 3D constitutive model for the full prediction of the behaviour of such NFMLs. Only one type of natural fibre composite was tested in this study. A comparative study is required in configuration with two or more natural fibre composites within the fibre metal laminates. Additionally, multiple alternative metal materials can also be applied, which can bring in more room for exploring a new combination of fibre-metal laminates. Lastly, the feasibility of applying natural fibre composite to the aerospace structural component is also worth investigating.

## Figures and Tables

**Table 1:** Isotropic hardening data for Aluminium 2024-T3.

Flow Stress (Mpa)	294	335	362	381	399	415	430	443	459	469	480
Plastic Strain (%)	0	1.1	2.2	3.2	4.4	5.5	6.7	7.8	9.2	10.1	11.2

**Table 2:** Material properties of the E-glass/Epoxy composite.

Mechanical Properties	Value
$\mathrm{Longitudinal}\;\mathrm{modulus}\;E_{1}$ (Mpa)	30,500
$\mathrm{Transverse}\;\mathrm{modulus}\;E_{2}$ (Mpa)	4030
$\mathrm{Major}\;\mathrm{Poisson}’s\;\mathrm{ratio}\;V_{12}$	0.29
$\mathrm{In}-\mathrm{plane}\;\mathrm{shear}\;\mathrm{modulus}\;G_{12}$ (Mpa)	2080
$\mathrm{Shear}\;\mathrm{modulus}\;G_{13}$ (Mpa)	2080
$\mathrm{Shear}\;\mathrm{modulus}\;G_{23}$ (Mpa)	1440
$\mathrm{Longitudinal}\;\mathrm{tensile}\;\mathrm{strength}\;X_{T}$ (Mpa)	700
$\mathrm{Longitudinal}\;\mathrm{compressive}\;\mathrm{strength}\;X_{C}$ (Mpa)	300
$\mathrm{Transverse}\;\mathrm{tensile}\;\mathrm{strength}\;Y_{T}$ (Mpa)	39
$\mathrm{Transverse}\;\mathrm{compressive}\;\mathrm{strength}\;Y_{C}$ (Mpa)	128
$\mathrm{Longitudinal}\;\mathrm{shear}\;\mathrm{strength}\;S_{12}$ (Mpa)	50
$\mathrm{Transverse}\;\mathrm{shear}\;\mathrm{strength}\;S_{23}$ (Mpa)	50
$\mathrm{Longitudinal}\;\mathrm{tensile}\;\mathrm{fracture}\;\mathrm{energy}\;G_{1t}$ (mJ)	92
$\mathrm{Longitudinal}\;\mathrm{compressive}\;\mathrm{fracture}\;\mathrm{energy}\;G_{1C}$ (mJ)	79
$\mathrm{Transverse}\;\mathrm{tensile}\;\mathrm{fracture}\;\mathrm{energy}\;G_{2t}$ (mJ)	0.22
$\mathrm{Transverse}\;\mathrm{compressive}\;\mathrm{fracture}\;\mathrm{energy}\;G_{2C}$ (mJ)	0.61

**Table 3:** Material properties of the flax/polypropylene.

Mechanical Properties	Value
$\mathrm{Longitudinal}\;\mathrm{modulus}\;E_{1}$ (Mpa)	22,980
$\mathrm{Transverse}\;\mathrm{modulus}\;E_{2}$ (Mpa)	3030
$\mathrm{Transverse}\;\mathrm{modulus}\;E_{3}$ (Mpa)	3030
$\mathrm{Major}\;\mathrm{Poisson}’s\;\mathrm{ratio}\;V_{12}$	0.38
$\mathrm{Poisson}’s\;\mathrm{ratio}\;V_{13}$	0.38
$\mathrm{Poisson}’s\;\mathrm{ratio}\;V_{23}$	0.7
$\mathrm{In}-\mathrm{plane}\;\mathrm{shear}\;\mathrm{modulus}\;G_{12}$ (Mpa)	1040
$\mathrm{Shear}\;\mathrm{modulus}\;G_{13}$ (Mpa)	1040
$\mathrm{Shear}\;\mathrm{modulus}\;G_{23}$ (Mpa)	1060
$\mathrm{Longitudinal}\;\mathrm{tensile}\;\mathrm{strength}\;X_{T}$ (Mpa)	334.85
$\mathrm{Longitudinal}\;\mathrm{compressive}\;\mathrm{strength}\;X_{C}$ (Mpa)	246.34
$\mathrm{Transverse}\;\mathrm{tensile}\;\mathrm{strength}\;Y_{T}$ (Mpa)	31.6
$\mathrm{Transverse}\;\mathrm{compressive}\;\mathrm{strength}\;Y_{C}$ (Mpa)	72.1
$\mathrm{Longitudinal}\;\mathrm{shear}\;\mathrm{strength}\;S_{12}$ (Mpa)	18.48
$\mathrm{Transverse}\;\mathrm{shear}\;\mathrm{strength}\;S_{23}$ (Mpa)	18.33
$\mathrm{Longitudinal}\;\mathrm{tensile}\;\mathrm{fracture}\;\mathrm{energy}\;G_{1t}$ (mJ)	80
$\mathrm{Longitudinal}\;\mathrm{compressive}\;\mathrm{fracture}\;\mathrm{energy}\;G_{1C}$ (mJ)	80
$\mathrm{Transverse}\;\mathrm{tensile}\;\mathrm{fracture}\;\mathrm{energy}\;G_{2t}$ (mJ)	0.2
$\mathrm{Transverse}\;\mathrm{compressive}\;\mathrm{fracture}\;\mathrm{energy}\;G_{2C}$ (mJ)	1

**Table 4:** Material properties of the cohesive interface.

Mechanical Properties	Value
$K_{n}=K_{s}=K_{t}$	$10^{6}\;N/{\mathrm{mm}}^{3}$
$t_{n}^{0}$	39 Mpa
$t_{s}^{0}$	50 Mpa
$t_{t}^{0}$	50 Mpa
$G_{IC}$	0.22 N/mm
$G_{IIC}$	0.36 N/mm
$G_{IIIC}$	0.36 N/mm
β	1.45

**Table 5:** Details of fibre metal laminates considered.

Laminate Code	Lay-Up	Fibre Type	Al Thickness (mm)	Flax/PP Thickness (mm)
A	[AL/0/0/AL/0/0/AL]	FLAX	0.4	0.625
B	[AL/0/90/AL/90/0/AL]	FLAX	0.4	0.625
C	[AL/+45/−45/AL/+45/−45/AL]	FLAX	0.4	0.625

## Data Availability

The data used in the manuscript can be found in the work of Aransáez Ortega, M. Numerical Simulation of the Impact Response of Natural Fibre Composites. 2019. Available online: https://trepo.tuni.fi/handle/123456789/27698. Any other data regarding the FE model can be requested by contacting the corresponding author Antigoni Barouni.
